# Notes and News

**Published:** 1905-01-07

**Authors:** 


### NOTES AND NEWS.

THE medical officer of health for Gorton is advocating the use of dried chrysanthemum petals as a substitute for tobacco.

MOST of the Powers have signed an international convention at the Hague exempting hospital ships from the payment of rates and dues in time of war.

THE Royal Hospital for Incurables and the Evelina Hospital for Children will benefit through the will of the late Mr. A. T. Yardley, of Clapham Common, who has constituted the two institutions residuary legatees of his estate.

COLONEL GILES, I.M.S., late Sanitary Commissioner for the South-West Province of India, and Dr. R. McConnell, of Canada, have been sent to the Gold Coast, Lagos, and Nigeria by the Liverpool School of Tropical Medicine to study the diseases peculiar to West Africa.

SINCE 1876 there has been a continuous increase of insanity in New Zealand. In the year referred to the proportion to population was 1·97 per 1,000, whilst in 1903 it had risen to 3·53 per 1,000. But the Inspector-General of Asylums in New Zealand does not attribute this to an increasing liability to insanity amongst the population, but to a variety of other reasons which are common to the growth of a young colony into a matured community.

A CANADIAN correspondent in the *Times* draws attention to a notable testimony in favour of vaccination. He states that 20 years ago smallpox was introduced into Montreal, where the French Canadian population were for the most part unvaccinated. This visitation carried off upwards of 3,000 victims. Since then the French inhabitants in Montreal have accepted vaccination, and, although the population has grown largely, only 38 deaths are recorded from small-pox since the disastrous outbreak of 1885.

PLAGUE has broken out at Port Florence in British East Africa. Four deaths are reported.

THE Hospital Saturday Fund collection of 1904 exceeded by £368 that of the previous year.

THE late Mr. John W. Lyell of Forest Hill has bequeathed £1,500 to the Middlesex Hospital, to produce an income for the Lyell Gold Medallist.

THE late Mrs. Emma Temple has bequeathed £2,000 each to the Charing Cross, St. George's, King's College, and the West London Hospitals.

THROUGH the will of the late Mrs. Ann Jameson of Sunderland the Sunderland Royal Infirmary and the Sunderland Eye Infirmary become residuary legatees of several thousand pounds.

ACCORDING to a statement in the *Times* an influential requisition is being signed among the Governors of St. Bartholomew's Hospital inviting Lord Ludlow to accept the office of Treasurer of that institution, in succession to Sir Trevor Lawrence, who is retiring.

AT the request of the Ameer of Afghanistan the Indian Government has selected Major H. Cleveland, of the Indian Medical Service, to take medical charge of the Ameer's household. Major Cleveland will still remain a member of the Indian Medical Service.

THE health of Jamaica is shown to have suffered severely from the effects of the cyclone of 1903. The death rate rose nearly 5 per cent., and the admissions into hospitals nearly 50 per cent. The insanitary condition is attributed to the pollution of the water through decaying matter being swept into the streams.

THE King's Hospital Fund will hold a Conference on February 2nd, to which the sixteen large hospitals are invited to send two representatives. The object of the Conference is to discuss the form of the report to be issued this year. A copy of the statistical report on the hospitals' expenditure referred to in the Prince of Wales's speech has been sent to each of the sixteen hospitals.

A DICKENS BALL is to take place in the Empress Rooms, Royal Palace Hotel, Kensington, on the 30th inst., in aid of the West Ham Hospital. One of the principal features will be a set of Lancers which will be danced during the evening by the grandchildren of the late Charles Dickens. The Ball is under the patronage of the Princess Christian and of other members of the Royal Family.

A COMMITTEE has been appointed to follow out the suggestions of Sir William Church and the proposals made at a recent meeting on the subject, to collect an endowment fund for a pathological department at the University of Oxford. The Rhodes trustees have guaranteed £200 per annum for five years towards the Readership held by Dr. Ritchie; and with Dr. Osler in the Chair of Medicine, medical study at the University may be expected to receive special stimulation.

